# The importance of thinking beyond the water-supply in cholera epidemics: A historical urban case-study

**DOI:** 10.1371/journal.pntd.0006103

**Published:** 2017-11-27

**Authors:** Matthew D. Phelps, Andrew S. Azman, Joseph A. Lewnard, Marina Antillón, Lone Simonsen, Viggo Andreasen, Peter K. M. Jensen, Virginia E. Pitzer

**Affiliations:** 1 Copenhagen Center for Disaster Research (COPE), Department of Public Health, Faculty of Health and Medical Sciences, Copenhagen, Denmark; 2 Department of Epidemiology, Johns Hopkins Bloomberg School of Public Health, Baltimore, Maryland, United States of America; 3 Center for Communicable Disease Dynamics, Harvard TH Chan School of Public Health, Boston, MA, United States of America; 4 Department of Epidemiology of Microbial Diseases, Yale School of Public Health, New Haven, Connecticut, United States of America; 5 Department of Science and the Environment, Roskilde University, Roskilde, Denmark; University of California San Diego School of Medicine, UNITED STATES

## Abstract

**Background:**

Planning interventions to respond to cholera epidemics requires an understanding of the major transmission routes. Interrupting short-cycle (household, foodborne) transmission may require different approaches as compared long-cycle (environmentally-mediated/waterborne) transmission. However, differentiating the relative contribution of short- and long-cycle routes has remained difficult, and most cholera outbreak control efforts focus on interrupting long-cycle transmission. Here we use high-resolution epidemiological and municipal infrastructure data from a cholera outbreak in 1853 Copenhagen to explore the relative contribution of short- and long-cycle transmission routes during a major urban epidemic.

**Methodology/Principal findings:**

We fit a spatially explicit time-series meta-population model to 6,552 physician-reported cholera cases from Copenhagen in 1853. We estimated the contribution of long-cycle waterborne transmission between neighborhoods using historical municipal water infrastructure data, fitting the force of infection from hydraulic flow, then comparing model performance. We found the epidemic was characterized by considerable transmission heterogeneity. Some neighborhoods acted as localized transmission hotspots, while other neighborhoods were less affected or important in driving the epidemic. We found little evidence to support long-cycle transmission between hydrologically-connected neighborhoods. Collectively, these findings suggest short-cycle transmission was significant.

**Conclusions/Significance:**

Spatially targeted cholera interventions, such as reactive vaccination or sanitation/hygiene campaigns in hotspot neighborhoods, would likely have been more effective in this epidemic than control measures aimed at interrupting long-cycle transmission, such as improving municipal water quality. We recommend public health planners consider programs aimed at interrupting short-cycle transmission as essential tools in the cholera control arsenal.

## Author summary

John Snow’s seminal work on the London cholera epidemic and Broadway pump helped establish cholera as a quintessential waterborne (long-cycle) pathogen. However, there is renewed interest in the role that short-cycle (e.g. food-borne and household) transmission plays in epidemic contexts. The distinction between transmission pathways can be important, as they may demand different interventions. However, disentangling these pathways requires high-quality epidemiological and contextual data that is rarely collected in outbreak situations. Here we use detailed cholera incidence and municipal infrastructure data from a cholera outbreak in 1853 Copenhagen to estimate the relative contributions of short- and long-cycle transmission to the epidemic. We find transmission between neighborhoods during the epidemic did not follow water pipe connections, suggesting little evidence of long-cycle transmission. Instead, we suggest that short-cycle transmission was likely critical to the propagation of the outbreak. Interventions targeting short-cycle transmission are important tools that merit further consideration by public health officials combating epidemic cholera.

## Introduction

Cholera transmission during an outbreak is known to occur via both ‘short-’ (for example, locally-mediated via food or household water) [1–3] and ‘long-cycles’ (environmentally-mediated via natural or manmade water and sanitation systems) [4,5]. Although long-cycle waterborne transmission is often considered the archetypical cholera transmission route [6,7], there is a growing interest in understanding the importance of short-cycle pathways [8–10]. Identifying the relative contributions of these different pathways has important public health implications for designing effective cholera interventions [11], yet remains difficult to ascertain from epidemiological data alone [12–14]. The proportion of cases infected via long-cycle relative to short-cycle transmission is probably highly context dependent, but the difficulty in distinguishing between the relative contributions of each route means that little data exists for any individual context [15,16]. The resulting uncertainty hinders planning of appropriate interventions. For example, spatially targeted interventions (e.g. targeted hygiene/sanitation or reactive vaccination programs) may be effective against short-cycle transmission [17], but suboptimal against long-cycle transmission [18].

To better characterize the relative importance of long-cycle vs short-cycle cholera transmission and mitigate parameter identifiability issues, high-resolution, high-quality epidemiological data can be augmented with data detailing the flow of drinking water in a specific setting. We use detailed data from an 1853 cholera outbreak in Copenhagen, Denmark as a case example. This outbreak has three key advantages from a modeling perspective: (1) this was likely an immunologically naive population as this was the first reported cholera outbreak in Copenhagen, (2) the outbreak was largely unmitigated by control measures as no effective treatments or interventions were implemented, and (3) historical datasets provide detailed information about the city’s hydraulic network.

The water supply of Copenhagen was composed of a network of hollowed wooden tree-trunks under low water-pressure, and thus vulnerable to outside contamination (S1 Fig). Additionally, there was no sewage system; rather, the street gutters functioned as light sewage drainage, with most human waste stored in unsealed cellars in each building and removed by night-men twice annually for use as fertilizer for food crops in nearby fields [19]. The piped drinking water was reported to be contaminated by seepage from these cesspools [20], thus elevating the risk of drinking contaminated water for downstream users during the cholera epidemic. Most piped water was sourced from nearby (<1 km) semi-artificial lakes, while some public fountains were supplied from water sources >10 km upstream [21].

Here, we use newly uncovered historical epidemiologic data from the 1853 epidemic along with modern statistical methods to investigate the relative contribution of short-cycle versus long-cycle transmission in a cholera outbreak. Fitting a time-series meta-population model to data from the 1853 cholera outbreak in Copenhagen, we characterize the spatio-temporal transmission dynamics and assess the signal of long-cycle environmentally-mediated transmission in the progression of the epidemic.

## Methods

### Data collection

Weekly cholera morbidity and mortality data for each city neighborhood and outlying communities were obtained from datasets compiled by contemporary physicians conducting active surveillance during the 1853 cholera epidemic in Copenhagen [22]. A corps of physicians traveled door-to-door to residences, hospitals (“sick-houses”), and “poor-houses” diagnosing and tabulating cases. Hospitalized cases were assigned to the neighborhood of residence, unless they were already in hospital prior to diagnosis, in which case they were geolocated to the hospital’s address. Six of the 13 neighborhoods were aggregated into two neighborhoods labeled “Combined upper” (Nørre and Klædebo) and “Combined lower” (Frimands, Strand, Snarens and Vester) due to low case counts and small geographic area. We excluded 621 (9%) cases that could not be geolocated, consisting of 122 (2%) cases from docked ships, and 499 (7%) from scattered outlying communities.

**Fig 1:**
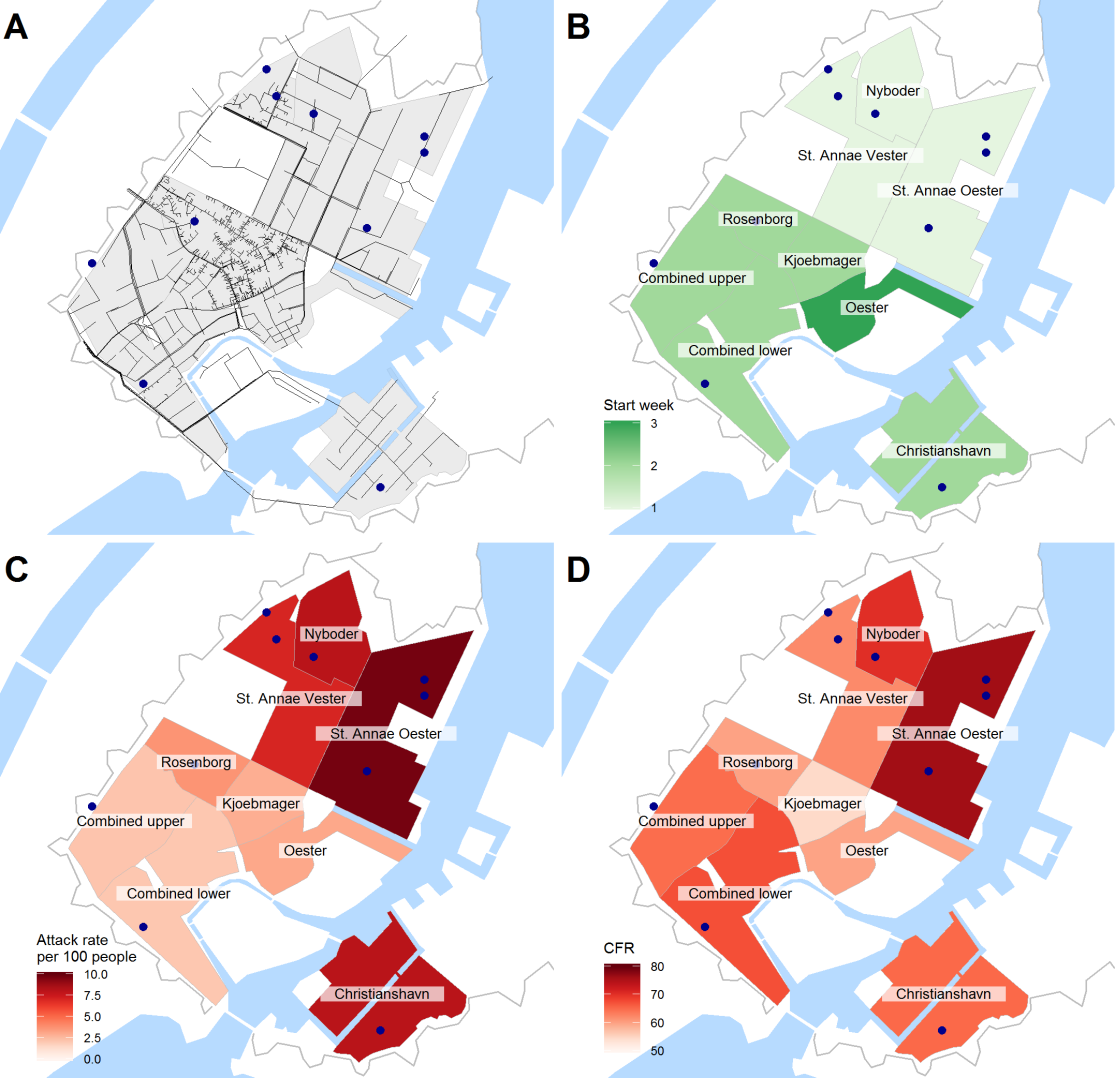
Maps of epidemic in Copenhagen, 1853. (A) The drinking-water pipe network. (B) The week number the first case was reported in each neighborhood. (C) The attack rate in each neighborhood. (D) The case-fatality rate in each neighborhood. The blue points represent hospitals, sick- and poor-houses. Map base layers and water-pipe data were digitized using QGIS from Nivellementskort over Kjöbenhavn. 1852. Archive reference: https://www.starbas.net/avmateriale.php?av_stam_id=46545.

Cholera cases were defined as patients with rice-water diarrhea and evidence of severe dehydration [23], making the historical diagnostic criteria stricter than the current WHO suspected cholera case definition [24]. Population size for each neighborhood was interpolated between the 1850 and 1855 census assuming a linear growth model. Population density was estimated for each neighborhood by georeferencing the neighborhoods using a Geographic Information System (GIS) and calculating area. Hydraulic data was digitized from a contemporary map [21] showing the layout and direction of flow of all water-pipes supplying drinking water for the city (Fig 1A). We created a binary asymmetric transition matrix of hydraulic connectivity describing the flow of water between neighborhoods, and a binary symmetric matrix describing neighborhoods that share a border (as a proxy for sewage runoff and human connectivity) (S1 Table and S2 Table). All GIS work was done in QGIS [25].

### Transmission models

To model the number of infectious people at each day, we fit a discrete time susceptible-infected-recovered (SIR) model to imputed daily case data We used the basic model structure from Azman et al. [17], with modifications as described below. In order to simulate daily case data (necessary because of the short generation interval for cholera), weekly cases were randomly reallocated to the seven days preceding the reported date. This reallocation was repeated 10 times to give 10 possible realizations of the epidemic. Each of the nine neighborhoods were considered as a discrete population. The model-predicted number of infected people in each neighborhood at each time-step was Poisson distributed where the mean was a function of the fraction susceptible in that neighborhood and a sum of the internal and external forces of infection.

**Fig 2:**
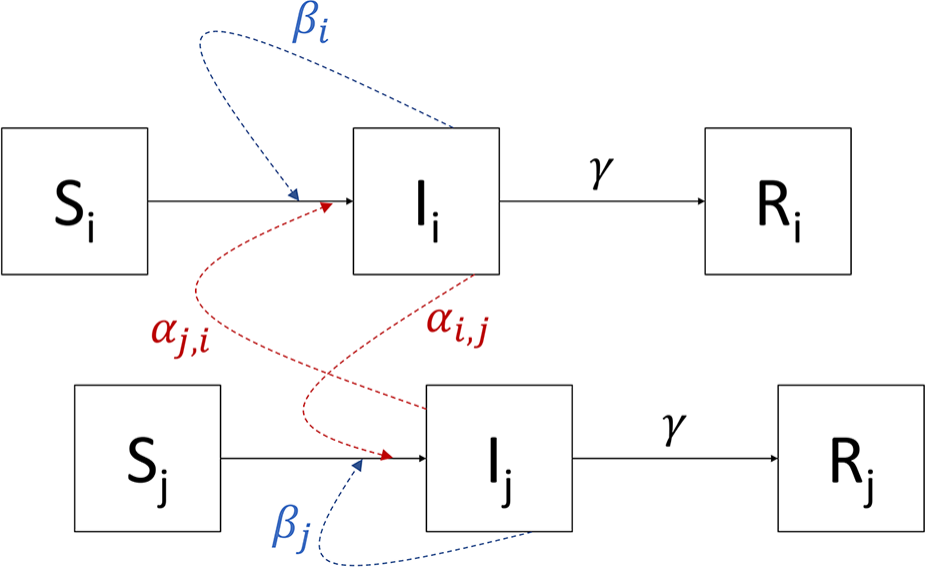
Diagram of transmission model structure. The boxes represent the different infection states in two neighborhoods, *i* and *j*. The solid arrows represent the different state transitions within the model, while the dotted lines represent how infectious individuals contribute to the force of infection in their own neighborhood (blue) and other neighborhoods (red).

We constructed a series of nested models in which the infection rate of susceptible individuals living in neighborhood *i*, or the force of infection, *λ*_*i*_, was the sum of an internal force of infection, *β*_*i*_, and an external force of infection from neighborhood *j* upon neighborhood *i*, *α*_*j*,*i*_ (Fig 2), such that:

$$\lambda_{i}=\beta_{i}I_{i}+\sum_{j\neq i}\alpha_{j,i}I_{j}$$

Each model had differing assumptions about *α* and *β*. Starting from the simplest base-case model to the most complex saturated model, we allowed: (1) a single *β* and single *α* for all neighborhoods such that *β*_*i*_ = *β* and *α*_*j*,*i*_ = *α*, (2) an individual *β*_*i*_ for each neighborhood and a single *α* for all neighborhoods such that *α*_*j*,*i*_ = *α*, and (3) an individual *β*_*i*_ for each neighborhood and an asymmetric *α*_*j*,*i*_ such that *α*_*j*,*i*_ ≠ *α*_*i*,*j*_ (S1 Text).

In order to estimate the effect of the water supply on the epidemic, we used two methods. First, we fit a linear regression model using the log of the median cross-neighborhood transmission coefficients (*α*_*j*,*i*_) from model 3 (saturated model) as the outcome and tested whether the hydraulic-transition and geographic-proximity variables were significant predictors of between-neighborhood transmission at a significance level of 0.05 (S1 Text, S1 Table and S2 Table). Second, we incorporated the hydraulic-connectivity and geographic-proximity matrices into model 2, producing models 2b and 2c, respectively. To assess the effect of hydraulic connectivity (model 2b), we allowed *α*_*j*,*i*_ to vary depending on whether the neighborhoods were connected via water pipes, such that:

$$\alpha_{j,i}=\left\{ \begin{array}{ll} \alpha_{0} & if\;no\;water\;connection\;j\to i\\ \alpha_{0}+\alpha_{1} & if\;water\;connection\;j\to i \end{array}\right.$$

To incorporate geographic proximity (model 2c), we added an additional term if the neighborhoods shared a border. The resulting *α*_*j*,*i*_ can be described as:

$$\alpha_{j,i}=\left\{ \begin{array}{ll} \alpha_{0} & if\;no\;shared\;border\;or\;water\;connection\;j\to i\\ \alpha_{0}+\alpha_{1} & if\;no\;shared\;border\;but\;water\;connection\;j\to i\\ \alpha_{0}+\alpha_{1}+\alpha_{2} & if\;shared\;border\;and\;water\;connection\;j\to i \end{array}\right.$$

Parameter estimation was done using Markov chain Monte Carlo (MCMC) methods in JAGS 4.2.0 [26] in R 3.3.1 [27]. Each model was run once on each of the 10 possible realizations of the imputed daily epidemic data. We used four MCMC chains with a burn-in of 40,000 iterations and sampled the subsequent 60,000 iterations. Chain convergence was assessed with a potential scale reduction factor $\left(\hat{R}\right)$ cutoff of 1.05 and a visual assessment of the trace plots. Posterior samples from all four chains and all 10 data realizations were pooled and summarized (S1 Text). Model preference was assessed using the average Watanabe-Akaike Information Criterion (WAIC); a difference of five in WAIC was considered significant, translating to a fold difference of *e*^5/2^ = 12.2 in terms of posterior probability on the model state space [28]. WAIC was chosen due to recent developments in this field suggesting WAIC is better able to capture the tradeoffs between model complexity and fit in a Bayesian framework than the more commonly used DIC [29,30].

We validated our model by simulating the outbreak in each neighborhood. To do so, we selected a parameter set from the joint posterior distribution and then drew from the number of cases at the next time step from the appropriate Poisson distribution in each neighborhood. Point-wise prediction intervals were constructed by taking the 2.5 and 97.5 percentiles at each time-step. We then used the simulated data to refit the model parameters to test if the model could recapture the original parameter estimates.

To assess the heterogeneity of transmission efficiency by neighborhood, we calculated the internal reproductive number (*R*_*int*_), the outflowing reproductive number (*R*_*out*_), the inflowing reproductive number (*R*_*in*_), and the total reproductive number (*R*_*tot*_) within each neighborhood using the force of infection coefficients. The *R*_*int*_, *R*_*out*_, and *R*_*tot*_ can be interpreted as the number of cases a single infectious case produces within its own neighborhood, in all other neighborhoods, and in the whole city respectively, while the R_in_ represents the number of cases produced within one neighborhood by a single infectious case in the rest of the city.

## Results

### Overview

**Fig 3:**
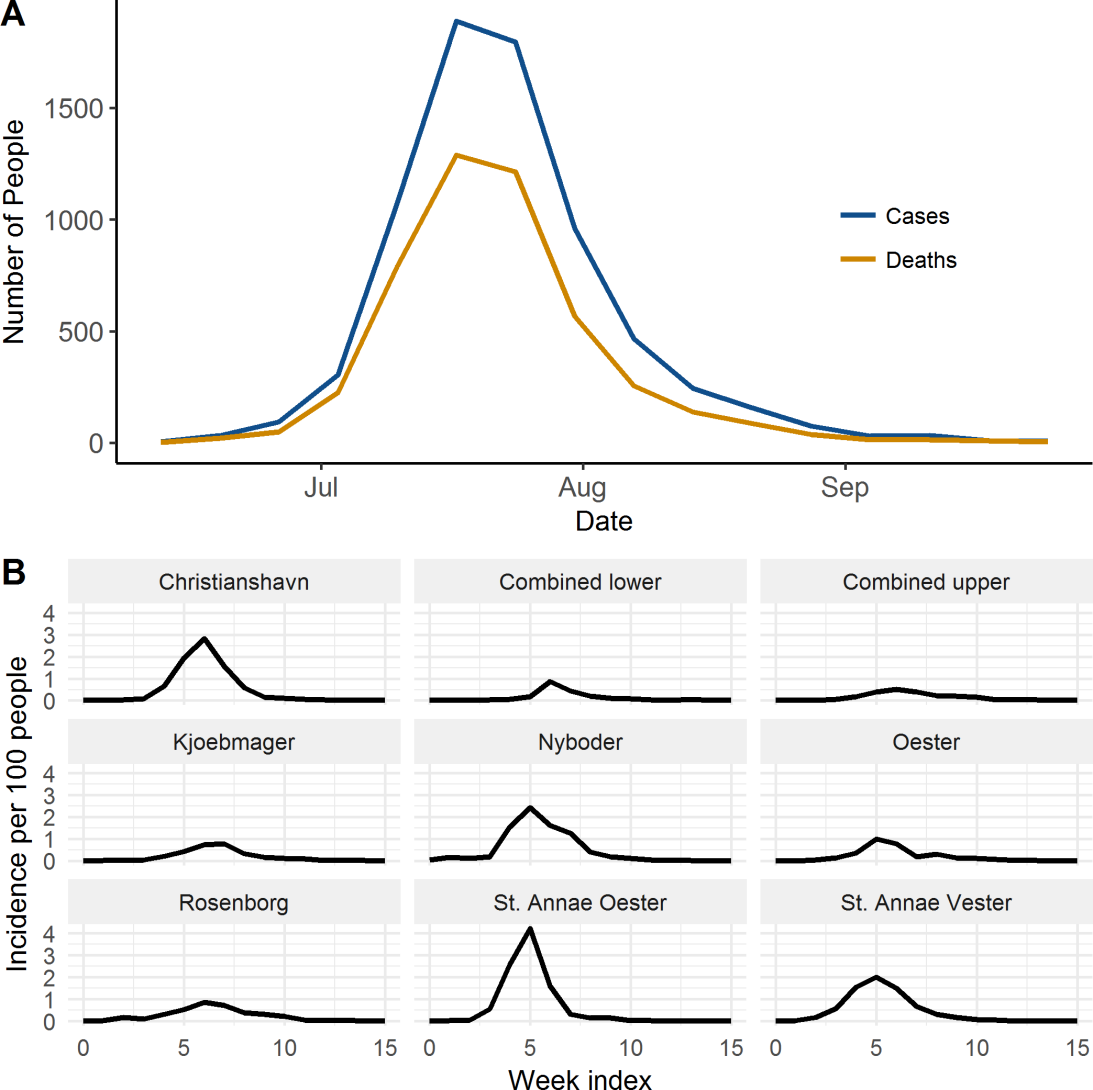
Time-series plot of epidemic. (A) At the city level. (B) In each neighborhood.

The epidemic began on June 12 with two reported cases in the Nyboder neighborhood among individuals working on (or in close contact with) ships. The epidemic soon spread to other neighborhoods (Fig 3A and 3B, S1 Dataset). The epidemic was declared over on October 1, although four cases were reported subsequently in October. A total of 7,173 cases were reported, of which 5,953 (83%) were community acquired and 1,220 (17%) were acquired in hospitals or poor-houses. A total of 4,717 died, resulting in a case fatality ratio (CFR) of 66%. The CFR among hospital/poor-house-acquired infections was 77%, as compared to 63% in community-acquired infections. A total of 6,552 cases (91%) occurred within the city walls and were included in this analysis.

The outbreak was spatially heterogeneous over the city, despite its small geographic size. Attack rates within the city walls ranged from 2.1 to 9.6 per 100, and the CFR ranged from 76% to 54% (Table 1, Fig 1C and 1D). The neighborhood attack rates were not associated with the neighborhood’s population density as assessed by a linear regression (p = 0.99).

**Table 1:** Overview of the epidemic in each neighborhood.

Quarter	Population	Cases	Deaths	Attack Rate (per 100)	CFR (%)
Christianshavn	15,836	1,278	832	8.1	65.1
Combined lower	20,663	431	290	2.1	67.3
Combined upper	15,991	354	229	2.2	64.7
Kjoebmager	11,544	340	185	2.9	54.4
Nyboder	7,195	583	410	8.1	70.3
Oester	8,553	268	160	3.1	59.7
Rosenborg	10,214	383	229	3.7	59.8
St. Annae Oester	11917	1154	882	9.7	76.4
St. Annae Vester	24655	1761	1091	7.1	62.0

### Model selection

**Fig 4:**
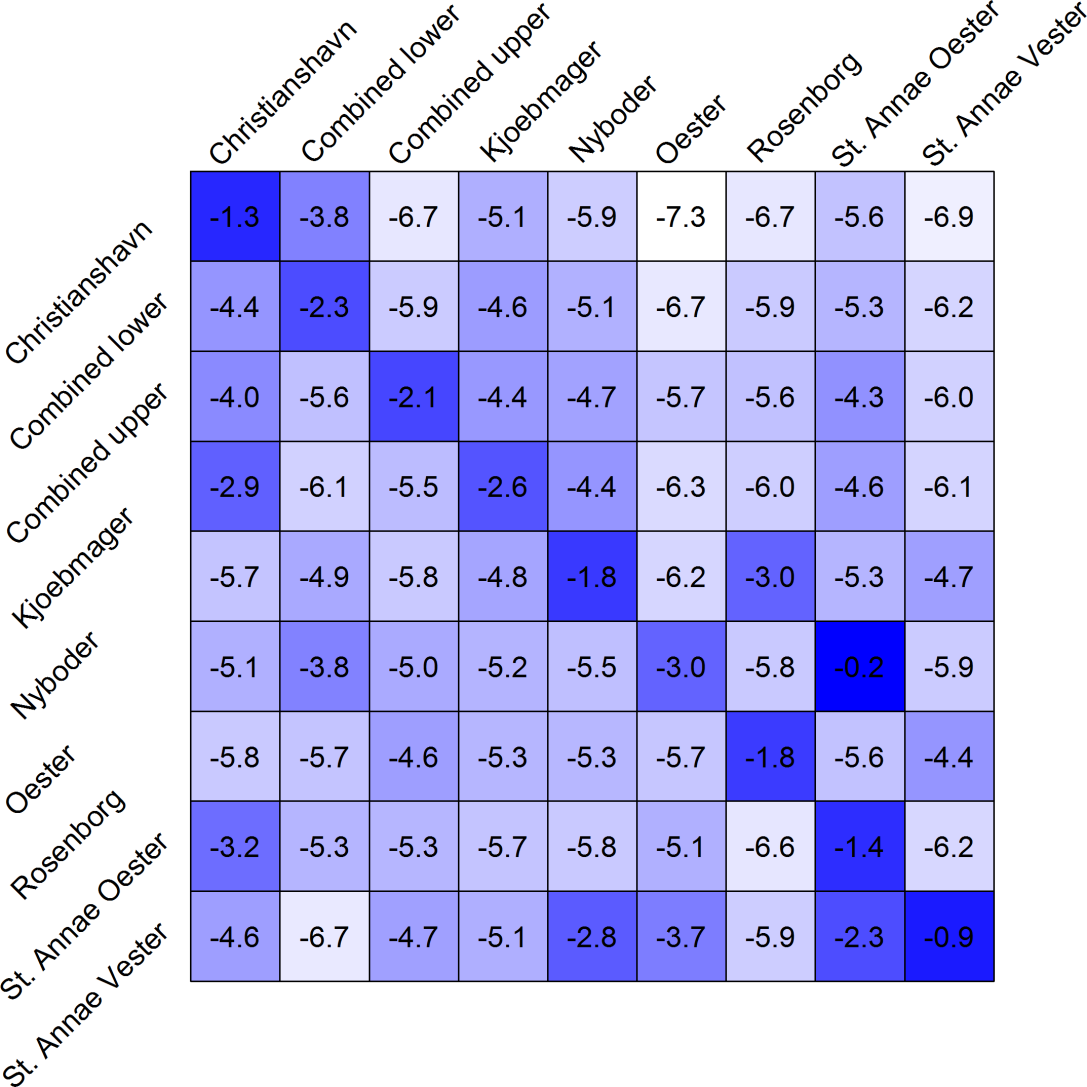
Median of the log of the transmission coefficients from model 3 (fully saturated model). The diagonals represent log(*β*_*i*_) and off-diagonals log(*α*_*j*,*i*_). For example, row 4 (Kjoebmager), column 1 (Christianshavn) can be read as the transmission coefficient for cases arising in Kjoebmager from cases in Christianshavn. Darker blue represents higher transmission efficiency.

Model selection carried out using WAIC (Table 2) indicated the best model allowed for asymmetric and heterogeneous transmission between neighborhoods (model 3); this heterogeneity could not be explained by hydraulic and geographic connectivity (model 2b and 2c). Similarly, the linear regression on the median of the cross-neighborhood transmission coefficients (*α*_*j*,*i*_) (Fig 4) from the fully saturated model (model 3) provided no support that a hydraulic connection between neighborhoods was significantly associated with the force of transmission between neighborhoods, even after controlling for geographic proximity (Table 3). The internal forces of transmission in each neighborhood were not strongly associated with the neighborhood’s population density (p = 0.11).

**Table 2:** WAIC values for each model (columns) for each of the 10 epidemic realizations (rows).

model 1	model 2	model 2b	model 2c	model 3
4,425	3,902	3,902	3,902	3,812
4,302	3,850	3,850	3,850	3,744
4,411	3,943	3,944	3,944	3,817
4,292	3,825	3,824	3,825	3,740
4,277	3,799	3,799	3,799	3,686
4,415	3,927	3,927	3,927	3,834
4,383	3,891	3,891	3,891	3,784
4,485	4,003	4,003	4,002	3,909
4,278	3,821	3,821	3,821	3,744

**Table 3:** Linear regression on the log of the median of between-neighborhood transmission coefficients.

Explanatory variable	Log effect size (95% CI)	p-value
Water connection	0.06 (-0.55, 0.68)	0.84
Shared border	0.14 (-.50, 0.78)	0.67

Based on the fully saturated model (model 3) with indicator variables for water-connections and shared-borders as explanatory variables.

### Simulating epidemics

**Fig 5:**
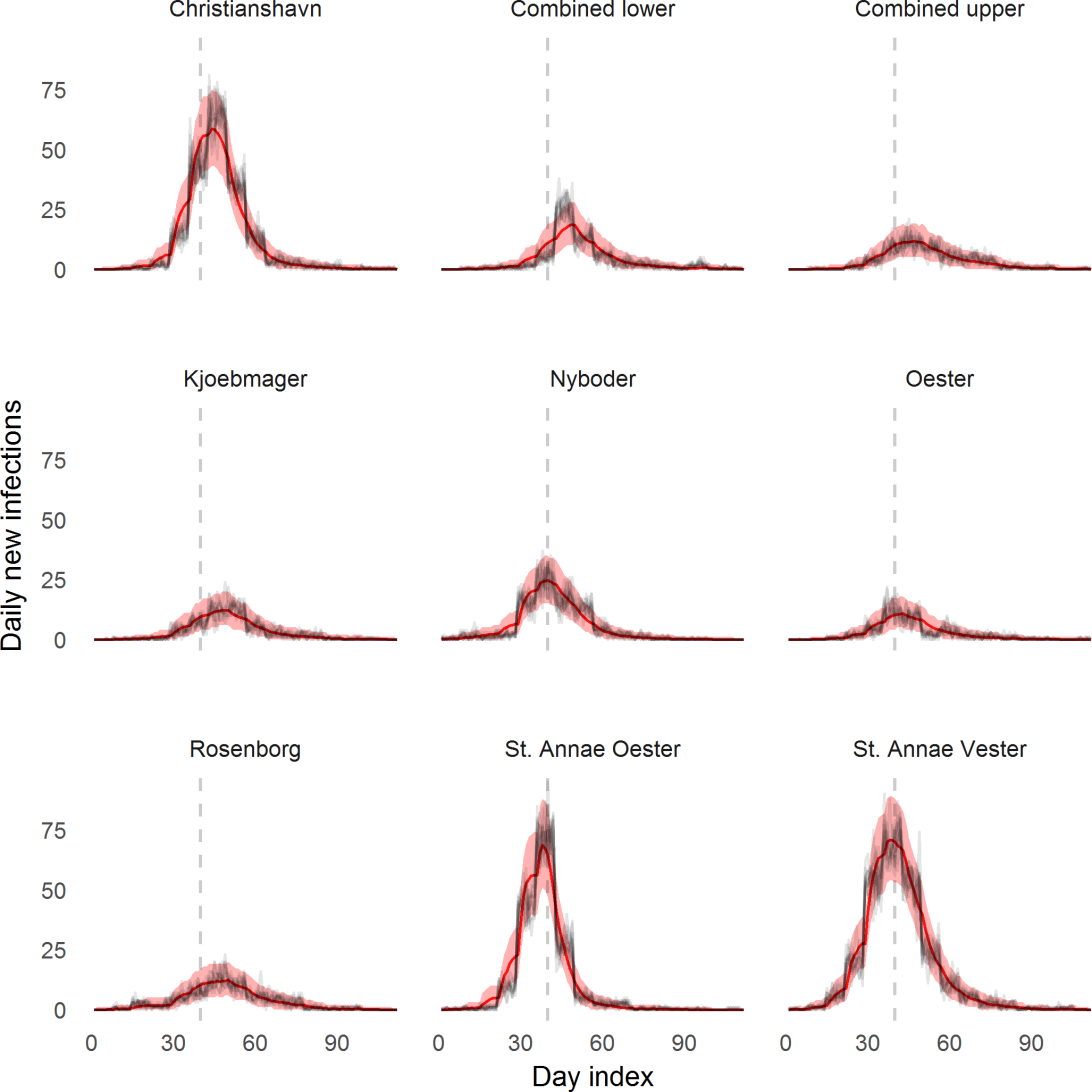
Result of one-step-ahead model predictions. The mean number of new infectious cases predicted one-step-ahead with 95% prediction intervals in red. The black lines represent the 10 realizations of the epidemic used as data for parameter fitting. The vertical dotted line represents day 40 for reference between graphs.

**Fig 6:**
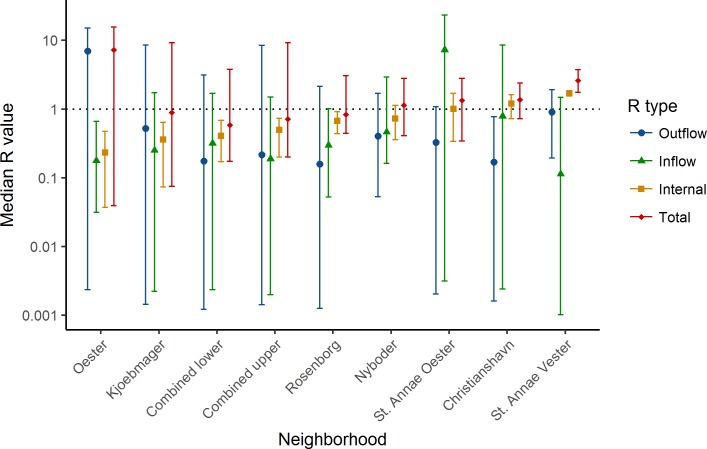
The median outflowing, inflowing, internal, and total reproductive numbers R_0_ for each quarter.

Using the fully saturated model (model 3), we simulated the outbreak in each neighborhood one day ahead, drawing a new parameter combination from the joint posterior distribution for each simulation (Fig 5). The *R*_*int*_ ranged from 0.2 (0.0–0.5) in Østerto to 1.7 (1.5–1.8) in St. Annæ Vester (Fig 6). One of the nine neighborhoods (St. Annæ Vester), had *R*_*int*_ >1, meaning it could maintain epidemics without infections from outside; in three other neighborhoods (St. Annæ Øster, Christianshavn and Nyboder), *R*_*int*_ was above 1 but the credible interval spanned 1.0, and in all remaining neighborhoods the *R*_*int*_ was below 1.0.

To validate the model, we re-estimated the model parameters from the simulated data for each of the 10 epidemic realizations. In seven of the 10 realizations, the 95% CI estimated from the simulated data overlapped 95% CI fitted from the original data for all 83 parameters. In the remaining three realizations overlap occurred in 82, 81 and 80 parameters respectively.

## Discussion

There is growing momentum towards re-thinking the dominance of long-cycle waterborne transmission in cholera outbreaks [31]. Using a spatially explicit metapopulation model, we captured the essential spatial-temporal dynamics of a major urban cholera epidemic in Copenhagen in 1853. Our analysis indicates that although transmission occurred between the different sections of the city, the data do not support an association between the trajectory of cases across neighborhoods and the flow of water/sewage from neighborhood to neighborhood. The lack of a signal of long-cycle transmission in the data, suggests the importance of short-cycle transmission.

The data do not match what would be expected for an epidemic primarily disseminated via long-cycle, waterborne transmission. There are three pieces of evidence for this: (1) we found the attack rates to be highly heterogeneous within the city despite all neighborhoods sharing common sources for drinking water and extensive water-pipe connections between the neighborhoods; (2) neighborhoods, such as Øster, Kjøbmager and Combined Lower, that were downstream of highly affected neighborhoods, such as St. Annæ Vester and Nyboder, and (3) a model fit to the between-neighborhood transmission coefficients from the fully saturated model (model 3) did not show evidence that water-flow between neighborhoods was associated with the force of infection between neighborhoods.

The transmission heterogeneity seen in Copenhagen has been documented in other cholera outbreaks over a range of spatial scales [17,32] and was unlikely to be confounded by socioeconomic status (SES). We suspect this because SES did not have a spatial structure at the city level; the rich and poor lived on the same city blocks with the rich living facing the streets and the poor inside interior courtyards [33]. Furthermore, population density was not associated with attack rate or the force of internal transmission. In terms of differential reporting, the relative uniformity of the case fatality ratio (Fig 1D) implies that all neighborhoods were similarly likely to report cases. This suggests that the transmission heterogeneity seen is a true phenomenon rather than an artifact of confounding.

The lack of support for long-cycle waterborne transmission in the Copenhagen epidemic has important public health implications for responding to present-day cholera outbreaks. Our analysis indicates that interrupting transmission by interventions targeting the centralized drinking water system would likely have had little effect. Despite the historical nature of our data, the Copenhagen outbreak can be a proxy for contemporary resource-constrained settings where water infrastructure is poor. Our results corroborate other research, both in historical and contemporary settings, where short-cycle transmission was described as a critical element in certain epidemic settings [1,10,31,34,35]. Taken together we propose public health practitioners need to more thoroughly investigate alternative transmission routes when investigating cholera outbreaks and planning interventions. A stronger focus on interrupting short-cycle transmission, including targeted reactive vaccination programs and sanitation and hygiene related interventions, could significantly reduce an epidemic’s impact [36–38].

There are several limitations to this analysis. Although we found no support for long-cycle transmission driving transmission between neighborhoods, our analysis could not rule out this transmission pathway entirely. Our results show some transmission did occur between neighborhoods, although it did not correlate with the piped water supply or neighborhood proximity (as a proxy for sewage run-off). Additionally, despite the high spatial-resolution of our data, long-cycle transmission within a single neighborhood is possible and would instead be captured by the within-neighborhood transmission coefficient (*β*_*i*_). It is probable that the epidemic resulted from multiple disparate transmission routes, a hypothesis consistent with recent models of the Haitian outbreak [34,39]. Our analysis could not investigate any specific alternative pathway, although contemporary reports of human waste used as fertilizer for food crops highlights one possible alternative route, which has been observed in other outbreaks as well [40].

Additionally, the available incidence data did not have the ideal temporal resolution for the analysis; it was aggregated into weekly time-steps, yet the generation time for cholera is suspected to be closer to 3–5 days [41]. To address this limitation, we randomly redistributed the weekly cases over the preceding week, thereby propagating uncertainty in the relationship among weekly-aggregated cases in terms of who transmitted to whom. In regard to hydraulic data, our measure of hydraulic connectivity was reduced to a binary value and does not fully capture the gradations of neighborhood water connections.

Although we argue the 1853 Copenhagen outbreak was not driven by long-cycle transmission, it may have played a larger role in outbreaks in other settings, at least in their initial stages [5,42–44]. In Haiti, for example, there is evidence that the initial outbreak, along with the continuing transmission, entailed significant long-cycle transmission based on hydrological transport of the pathogen [4,35,45,46]. The factors determining which mode of transmission will dominate new epidemics are not understood, but perhaps consists of a combination of cultural and environmental factors. The ability to classify the dominant transmission mode early on in an epidemic will be key to enacting effective contagion control strategies.

Using a unique and highly detailed dataset, we have shown that that long-cycle, environmentally-mediated transmission is not a prerequisite for explosive, large-scale cholera outbreaks. While an exact quantification of each pathway’s contribution remains difficult, our findings—taken together with previous research [10,17,31,35,36,38]—suggest spatially-targeted cholera interventions, such as reactive vaccination and hygiene/sanitation programs, are important tools to combat epidemics with significant short-cycle transmission. Moreover, programs targeting long-cycle waterborne transmission may not be effective in all outbreak settings.

## Supporting information

S1 Fig Example of Copenhagen municipal water-pipe infrastructure from around the time period of the epidemic. Photo by authors.
(TIFF)

S1 Table Matrix of water-pipe connections between neighborhoods. Going by columns, a “1” denotes that the neighborhood in column *j* receives water that has passed through the neighborhood in row *i*.
(PDF)

S2 Table Matrix of shared borders between neighborhoods. (PDF)

S1 Text Supplemental text. (PDF)

S1 Dataset Weekly cholera morbidity and mortality for each neighborhood in Copenhagen, 1853. (CSV)
