# Supplementary figures and images for: The importance of thinking beyond the water-supply in cholera epidemics: A historical urban case-study
Source: PLoS Negl Trop Dis. 2017 Nov 27;11(11):e0006103. doi: 10.1371/journal.pntd.0006103 (PMC5720805; doi:10.1371/journal.pntd.0006103)

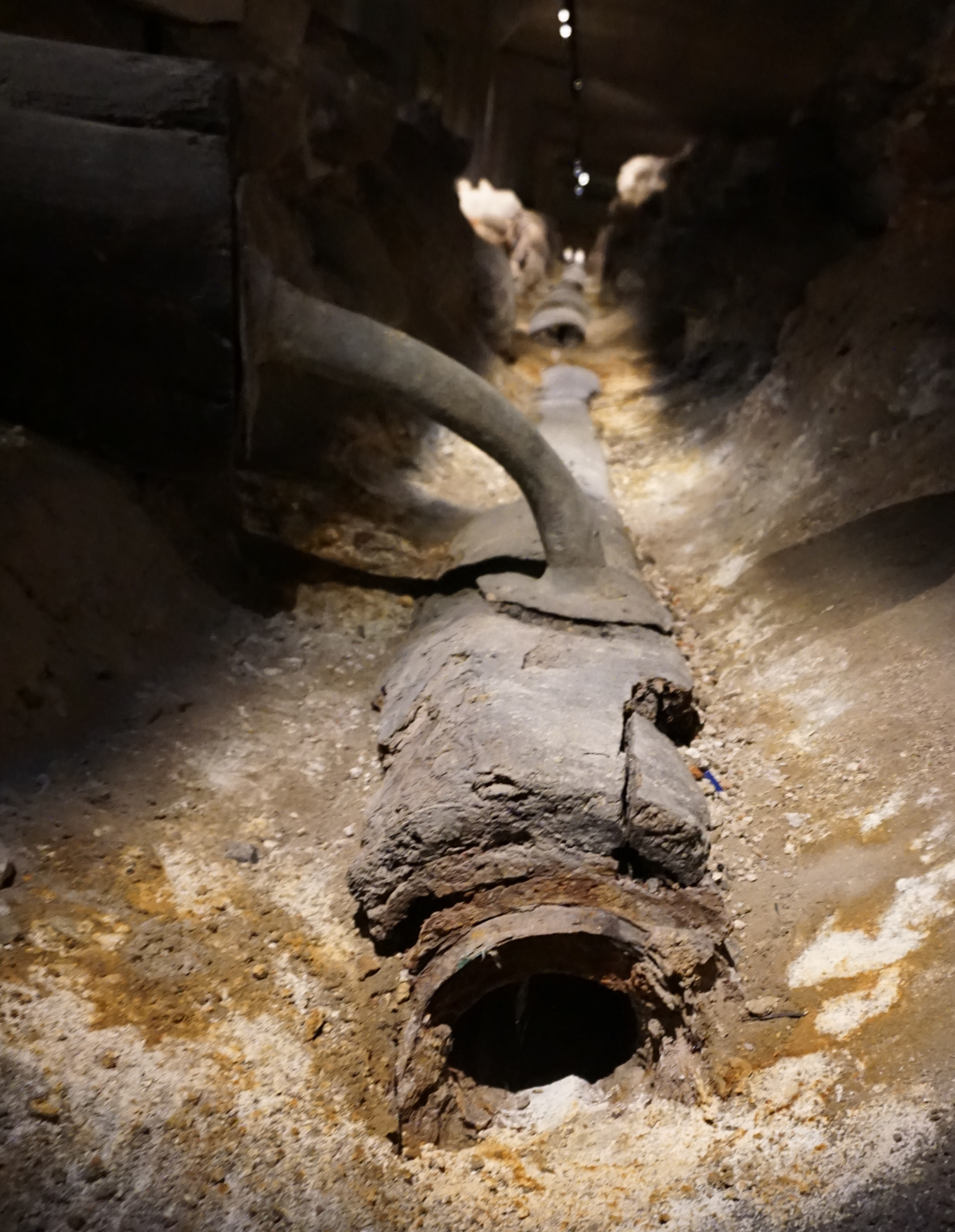

Supplement: S1 Fig — Photo by authors. (TIFF) [file pntd.0006103.s001.tiff]
