# Supplementary material for: The importance of thinking beyond the water-supply in cholera epidemics: A historical urban case-study
Source: PLoS Negl Trop Dis. 2017 Nov 27;11(11):e0006103. doi: 10.1371/journal.pntd.0006103 (PMC5720805; doi:10.1371/journal.pntd.0006103)
Supplement: S1 Table — Going by columns, a “1” denotes that the neighborhood in column j receives water that has passed through the neighborhood in row i. (PDF) [file pntd.0006103.s002.pdf]

Quarter	Christianshavn	Combined lower	Combined upper	Kjoebmager	Nyboder	Oester	Rosenborg	St. Annae Oester	St. Annae Vester
Christianshavn	NA	0	0	0	0	0	0	0	0
Combined lower	1	NA	0	1	0	1	0	0	0
Combined upper	0	1	NA	1	0	1	1	0	0
Kjoebmager	0	1	0	NA	0	1	1	1	0
Nyboder	0	1	0	1	NA	1	0	1	0
Oester	0	1	0	1	0	NA	0	0	0
Rosenborg	0	1	0	1	0	1	NA	0	0
St. Annae Oester	0	1	0	1	0	1	0	NA	0
St. Annae Vester	0	1	0	1	1	1	0	1	NA
