# Supplementary material for: The importance of thinking beyond the water-supply in cholera epidemics: A historical urban case-study
Source: PLoS Negl Trop Dis. 2017 Nov 27;11(11):e0006103. doi: 10.1371/journal.pntd.0006103 (PMC5720805; doi:10.1371/journal.pntd.0006103)
Supplement: S2 Table — (PDF) [file pntd.0006103.s003.pdf]

Quarter	Christianshavn	Combined lower	Combined upper	Kjoebmager	Nyboder	Oester	Rosenborg	St. Annae Oester	St. Annae Vester
Christianshavn	NA	0	0	0	0	0	0	0	0
Combined lower	0	NA	1	1	0	0	1	0	0
Combined upper	0	1	NA	0	0	0	1	0	0
Kjoebmager	0	1	0	NA	0	1	1	0	1
Nyboder	0	0	0	0	NA	0	0	1	1
Oester	0	1	0	1	0	NA	0	0	0
Rosenborg	0	1	1	1	0	0	NA	0	1
St. Annae Oester	0	0	0	0	1	0	0	NA	1
St. Annae Vester	0	0	0	1	1	0	1	1	NA
