# Supplementary material for: The importance of thinking beyond the water-supply in cholera epidemics: A historical urban case-study
Source: PLoS Negl Trop Dis. 2017 Nov 27;11(11):e0006103. doi: 10.1371/journal.pntd.0006103 (PMC5720805; doi:10.1371/journal.pntd.0006103)
Supplement: S1 Text — (PDF) [file pntd.0006103.s004.pdf]

The importance of thinking beyond the water-supply in cholera epidemics: a historical urban case-study

Matthew D. Phelps, Andrew S. Azman, Joseph A. Lewnard, Marina Antillón, Lone Simonsen, Viggo Andreasen, Peter K.M. Jensen, Virginia E. Pitzer.

S1 Supplemental Text

1.1 Model structure

We constructed a series of nested models where the force of infection acting upon neighborhood i was the sum of an internal force of infection, β_i and external force of infection from neigborhood j upon neighborhood i, $\alpha_{j,i}$.

From simple to complex we allowed (1) a single β and single α for all neighborhoods, such that $\beta_i = \beta$ and $\alpha_{j,i} = \alpha$. (2) An individual β_i for each neighborhood and a single α for all neighborhoods, such that $\alpha_{j,i} = \alpha$. (3) An individual β_i for each neighborhood and a single asymmetric $\alpha_{i,j}$ for each neighborhood pair, such that $\alpha_{j,i} \neq \alpha_{i,j}$.

Two additional models were constructed that were not reported in the main text. In model 2.1 allowed an individual β_i and α_i for each neighborhood, such that $\alpha_{j,i} = \alpha_i$. In model 2.2 we allowed an individual β_i for each neighborhood and symmetric $\alpha_{j,i}$ for each neighborhood pair, such that $\alpha_{j,i} = \alpha_{i,j}$. These models were not supported by the model selection process.

Each model was based upon the following construction:

$$^{new}\mathrm{I}_{i,t+1} \sim Poisson\left(\frac{S_{i,t}\phi}{N_i}(\beta_i I_{i,t} + \sum_{j\neq i}\alpha_{j,i}I_{j,t})\right)$$

where:
$^{new}\mathrm{I}_{i,t}$ = the number of reported new infectious cases in each neighborhood i at time t
$I_{i,t}$ = the total number of infectious cases in each neighborhood i at time t
$S_{i,t}$ = the number of susceptible people in each neighborhood i at time t
N_i = the total population of neighborhood i
β_i = the force of internal infection in neighborhood i
$\alpha_{j,i}$ = the force of infection from neighborhood j to neighborhood i.
ϕ = the fraction of cases that are reported

The total number of cases $I_{i,t}$ was updated via:

$$I_{i,t+1} = I_{i,t} + \frac{^{new}\mathrm{I}_{i,t}}{\phi} - R_{i,t}$$

where $R_{i,t}$ = the number of people who recovered or died from infection.

The number of recovered individuals $R_{i,t}$ was updated via:

$$R_{i,t+1} = \gamma I_{i,t}$$

where $\frac{1}{\gamma}$ = the duration of infectiousness.

The number of susceptible $S_{i,t}$ was updated via:

$$S_{i,t+1} = S_{i,t} - \frac{^{new}\mathrm{I}_{i,t}}{\phi}$$

The full system of model equations is thus:

$$S_{i,t+1} = S_{i,t} - \frac{^{new}\mathrm{I}_{i,t}}{\phi}$$

$$I_{i,t+1} = I_{i,t} + \frac{^{new}\mathrm{I}_{i,t}}{\phi} - R_{i,t}$$

$$R_{i,t+1} = \gamma I_{i,t}$$

1.2 Hydraulic connectivity and geographic proximity

To assess the effect of hydraulic connectivity we used two methods: (A) a linear regression, and (B) incorporating hydraulic and geographic connectivity into the meta-population model.

In method (A) we fit a linear model to the median of the log of the cross-neighborhood transmission coefficients ($\alpha_{j,i}$) from the fully saturated model (model 3) using the hydraulic transition matrix and geographic proximity matrix as covariates. The model can be written as follows:

$$y = \beta_0 + \beta_1 x_1 + \beta_2 x_2$$

where y is a vector of the median of the log of the cross neighborhood transmission coefficients ($\alpha_{i,j}$) and x_1 is a vector of hydraulic connectivity (Table S1) defined as

$$x_1 \begin{cases} 0 & \text{if no water connection exists } j \to i \\ 1 & \text{if water connection exists} j \to i \end{cases}$$

and x_2 is a vector of geographic proximity (Table S2) defined as

$$x_2 \begin{cases} 0 & \text{if no shared border exists } j \to i \\ 1 & \text{if shared border exists } j \to i \end{cases}$$

In method (B) we expanded model 2 to allow the force of infection (α) between two neighborhoods to vary depending on if the neighborhoods are connected via water pipes such that

$$\alpha_{j,i} \begin{cases} \alpha_0 & \text{if no water connection } j \to i \\ \alpha_0 + \alpha_1 & \text{if water connection } j \to i \end{cases}$$

creating model 2b. We then expanded model 2b to incorporate geographic proximity (model 2c) by adding an additional term α_2 if the neighborhoods shared a border. The resulting $\alpha_{i,j}$ can be described as

$$\alpha_{j,i} \begin{cases} \alpha_0 & \text{if no shared border or water connection } j \to i \\ \alpha_0 + \alpha_1 & \text{if no shared border but water connection } j \to i \text{ exists} \\ \alpha_0 + \alpha_1 + \alpha_2 & \text{if shared border and water connection } j \to i \text{ exists} \end{cases}$$

The effect of water, α_1, and the effect of the shared border, α_2, are not fitted to each neighborhood, but are shared citywide.

1.3 Model fitting

The model used in the paper (model 3) was fit using `JAGS 3.4` and the `runjags` and `rjags` libraries in R. The model priors were specified as thus:

$$^{new}\mathrm{I}_{i,t+1} \sim Poisson\left(\frac{S_{i,t}\phi}{N_i}(\beta_i I_{i,t} + \sum_{j \neq i} \alpha_{j,i} I_{j,t})\right)$$

$$log(\alpha_{j,i}) \sim N(\mu_1, \tau_1)$$

$$log(\beta_i) \sim N(\mu_2, \tau_2)$$

$$\mu_1 \sim N(0, \frac{1}{0.001})$$

$$\mu_2 \sim N(0, \frac{1}{0.001})$$

$$\tau_1 \sim \Gamma(0.001, 0.001)$$

$$\tau_2 \sim \Gamma(0.001, 0.001)$$

$$logit(\phi) \sim N(0, \frac{1}{0.001})$$

$$\gamma \sim exp(5)$$

The Gamma distribution for τ_1 and τ_2 was parameterized in terms of shape and rate. The exponential distribution for γ was parameterized in terms of a rate.

1.4 Model selection

We used the Watanabe-Akaike information criterion (WAIC) for model selection where a difference of at least 5 was considered significant. Note models 2.1 and 2.2 are not reported in the main text.

model 1	model 2	model 2b	model 2c	model 2.1	model 2.2	model 3
4425	3902	3902	3902	3871	3846	3812
4302	3850	3850	3850	3832	3802	3744
4411	3943	3944	3944	3915	3891	3817
4292	3825	3824	3825	3806	3773	3740
4277	3799	3799	3799	3786	3744	3686
4415	3927	3927	3927	3909	3873	3834
4383	3891	3891	3891	3862	3834	3784
4485	4003	4003	4002	3976	3931	3909
4278	3821	3821	3821	3807	3767	3744
4504	3997	3997	3997	3980	3957	3920

For every realization of the epidemic, model 3 performed the best.

1.5 Posterior summary statistics

For the selected model, model 3 (fully saturated model), we calculated the median and standard deviation of the posterior distribution for all fitted parameters. The posterior median and standard deviation of $log(\beta_i)$ and $log(\alpha_{j,i})$ are in figure 1 and figure 2 respectively.

The posterior median (standard deviation) for ϕ was 0.0987 (0.0011).
The posterior median (standard deviation) for γ was 0.2445 (0.0196)

Posterior median of $\log(\beta_i)$ and $\log(\alpha_{j,i})$

	Christianshavn	Combined_lower	Combined_upper	Kjoebmager	Nyboder	Oester	Rosenborg	St. Annae Oester	St. Annae Vester
Christianshavn	−1.278	−3.793	−6.677	−5.108	−5.950	−7.340	−6.672	−5.635	−6.890
Combined_lower	−4.373	−2.311	−5.894	−4.563	−5.142	−6.695	−5.859	−5.274	−6.166
Combined_upper	−4.046	−5.568	−2.148	−4.436	−4.740	−5.702	−5.592	−4.260	−6.049
Kjoebmager	−2.888	−6.059	−5.473	−2.552	−4.352	−6.296	−5.967	−4.626	−6.126
Nyboder	−5.675	−4.922	−5.818	−4.822	−1.778	−6.196	−2.967	−5.255	−4.654
Oester	−5.124	−3.836	−4.966	−5.232	−5.531	−2.973	−5.829	−0.184	−5.884
Rosenborg	−5.819	−5.695	−4.630	−5.287	−5.333	−5.735	−1.831	−5.639	−4.351
St. Annae Oester	−3.238	−5.274	−5.285	−5.685	−5.835	−5.124	−6.642	−1.449	−6.230
St. Annae Vester	−4.624	−6.726	−4.660	−5.086	−2.752	−3.696	−5.943	−2.336	−0.914

Figure 1: The posterior medians for $log(\beta_i)$ (diagonal) and $log(\alpha_{j,i})$ (off-diagonal) for each neighborhood from model 3. For example, row 4 (Kjoebmager), column 1 (Christianshavn) can be read as the posterior median of the log transmission coefficient for cases arising in Kjoebmager from cases in Christianshavn

Posterior standard deviation of $\log(\beta_i)$ and $\log(\alpha_{j,i})$

	Christianshavn	Combined_lower	Combined_upper	Kjoebmager	Nyboder	Oester	Rosenborg	St. Annae Oester	St. Annae Vester
Christianshavn	0.3332	1.7675	1.7690	1.8646	1.9757	1.6579	1.7817	2.2406	1.7663
Combined_lower	2.6137	0.4775	1.9951	2.0896	2.2472	1.7949	2.0054	2.4305	1.9654
Combined_upper	2.7533	2.1072	0.5964	2.1897	2.3734	1.9791	2.1098	3.0609	2.0089
Kjoebmager	2.9119	1.9965	2.1267	0.8095	2.4317	1.8924	2.0076	2.9529	1.9879
Nyboder	2.0792	1.8114	1.9270	2.0276	0.3651	1.9055	0.8288	2.1431	2.2372
Oester	2.5301	2.3251	2.3326	2.2205	2.2727	0.8719	2.0470	3.4616	2.0756
Rosenborg	2.0838	2.0355	2.1908	2.1344	2.2438	2.0029	0.2552	2.2473	2.3944
St. Annae Oester	2.0041	1.8002	1.8115	1.8419	2.0562	1.9192	1.7768	0.4908	1.9310
St. Annae Vester	2.0710	1.7461	1.5261	1.5926	0.5522	0.8784	1.8145	1.9425	0.0714

Figure 2: The posterior standard deviations for $log(\beta_i)$ (diagonal) and $log(\alpha_{j,i})$ (off-diagonal) for each neighborhood from model 3.
